# Characterization of Multiple Cytokine Combinations and TGF-β on Differentiation and Functions of Myeloid-Derived Suppressor Cells

**DOI:** 10.3390/ijms19030869

**Published:** 2018-03-15

**Authors:** Cho-Rong Lee, Wongeun Lee, Steve K. Cho, Sung-Gyoo Park

**Affiliations:** School of Life Sciences, Gwangju Institute of Science and Technology (GIST), Gwangju 61005, Korea; seada872@gmail.com (C.-R.L.); wodrl1234@gist.ac.kr (W.L.); scho@gist.ac.kr (S.K.C.)

**Keywords:** myeloid-derived suppressor cells, transforming growth factor-β, granulocyte-macrophage colony stimulating factor, granulocyte colony stimulating factor, interleukin-4, interleukin-6

## Abstract

Myeloid-derived suppressor cells (MDSCs) regulate T cell immunity, and this population is a new therapeutic target for immune regulation. A previous study showed that transforming growth factor-β (TGF-β) is involved in controlling MDSC differentiation and immunoregulatory function in vivo. However, the direct effect of TGF-β on MDSCs with various cytokines has not previously been tested. Thus, we examined the effect of various cytokine combinations with TGF-β on MDSCs derived from bone marrow cells. The data show that different cytokine combinations affect the differentiation and immunosuppressive functions of MDSCs in different ways. In the presence of TGF-β, interleukin-6 (IL-6) was the most potent enhancer of MDSC function, whereas granulocyte colony-stimulating factors (G-CSF) was the most potent in the absence of TGF-β. In addition, IL-4 maintained MDSCs in an immature state with an increased expression of arginase 1 (Arg1). However, regardless of the cytokine combinations, TGF-β increased expansion of the monocytic MDSC (Mo-MDSC) population, expression of immunosuppressive molecules by MDSCs, and the ability of MDSCs to suppress CD4^+^ T cell proliferation. Thus, although different cytokine combinations affected the MDSCs in different ways, TGF-β directly affects monocytic-MDSCs (Mo-MDSCs) expansion and MDSCs functions.

## 1. Introduction

Myeloid-derived suppressor cells (MDSCs) are a heterogeneous cell population of myeloid-derived cells comprising immature myeloid cells (i.e., immature macrophages, granulocytes, dendritic cells, and other myeloid cells in the early stages of differentiation) [1]. MDSCs have immunosuppressive effects; murine MDSCs are commonly defined by expression of CD11b and Gr-1 and are present in low numbers in the spleen and blood of tumor-free mice [2]. However, in cases of inflammatory disease (e.g., cancer, infection, sepsis, and trauma), MDSCs expand and accumulate in the spleen, blood, lymph nodes, and tissues [3]. Murine MDSCs are characterized according to two epitopes of Gr-1, Ly-6G, and Ly-6C, which are detected by specific antibodies [3]. These epitopes allow separation of MDSCs into two different subpopulations: granulocytic-MDSCs (Gr-MDSCs; CD11b^+^Ly-6G^high^Ly-6C^low^) and monocytic-MDSCs (Mo-MDSCs; CD11b^+^Ly-6G^dim^Ly-6C^high^) [4]. Each subpopulation suppresses T cell proliferation via a different mechanism. Gr-MDSCs secrete high levels of reactive oxygen species (ROS) and show increased arginase 1 (Arg1) activity, whereas Mo-MDSCs express low levels of Arg1 but secrete large amounts of nitric oxide (NO) via inducible nitric oxide synthase (iNOS) [3,5,6]. Mo-MDSCs also secrete transforming growth factor-β (TGF-β) and interleukin-10 (IL-10), which have direct immunosuppressive effects and induce regulatory T cells, which suppress tumor-specific T cell responses [7].

Colony-stimulating factors (CSFs) comprise granulocyte-macrophage CSF (GM-CSF), macrophage CSF (M-CSF), and granulocyte CSF (G-CSF), all of which regulate myeloid cell numbers and function under steady state and inflammatory conditions [8]. Clinically, GM-CSF is used as a vaccine adjuvant to induce T cell-mediated anti-tumor immune responses [9]. However, high-dose GM-CSF vaccines may impair the immune response by generating MDSCs in vivo [10]. GM-CSF is the main factor that drives MDSC differentiation from the bone marrow [3,11]. GM-CSF and G-CSF maintain the survival of differentiated myeloid cells [12]. G-CSF also regulates neutrophil differentiation and migration from bone marrow to blood [13]. G-CSF increases the survival and activation of MDSCs via the signal transducer and activator of transcription 3 (STAT3) signaling pathway [14]. Tumor-induced CD11b^+^IL-4Rα^+^-expressing monocytes have immunosuppressive effects on T cells [15]. Interleukin-4 receptor α (IL-4Rα) is the receptor for both IL-4 and IL-13 [16] and is also a biomarker of immunosuppressive MDSCs [17,18]. MDSCs show increased Arg1 activity [19] and enhanced immunosuppressive properties upon exposure to IL-4 [20]. A previous study showed that IL-6 produced by mammary carcinoma cells caused MDSCs to accumulate and restore tumor progression by acting as a downstream mediator of IL-1β [21]. Also, IL-6 activates the STAT3 signaling pathway to increase the number of MDSCs [22]. In addition, a recent study showed that TGF-β affects the MDSCs differentiation and functions [23].

Here, we characterized MDSCs induced by a combination of six different cytokines, with or without TGF-β. The selected cytokines are known to be involved in controlling MDSCs as described above. Although the effect of TGF-β on MDSCs has recently been characterized, the combinational effect has not previously been tested. Thus, we checked the effect of the cytokine combinations on MDSCs. In our experiments, MDSCs generated in the presence of TGF-β showed higher expression of immunosuppressive molecules and higher suppressive activity for CD4^+^ T cell proliferation. IL-4 maintained MDSCs in an immature state. IL-6 increased the immunosuppressive effects of MDSCs induced with TGF-β while G-CSF was the most potent driver of MDSC function induced without TGF-β. Overall, although each cytokine differentially regulates MDSCs differentiation and function, the TGF-β induces Mo-MDSC expansion and increases MDSCs function in all differentiation cases.

## 2. Results

### 2.1. Characterization of Subpopulation in the Differentiated MDSCs Induced by Different Cytokine Combinations with or without TGF-β

**Figure 1:**
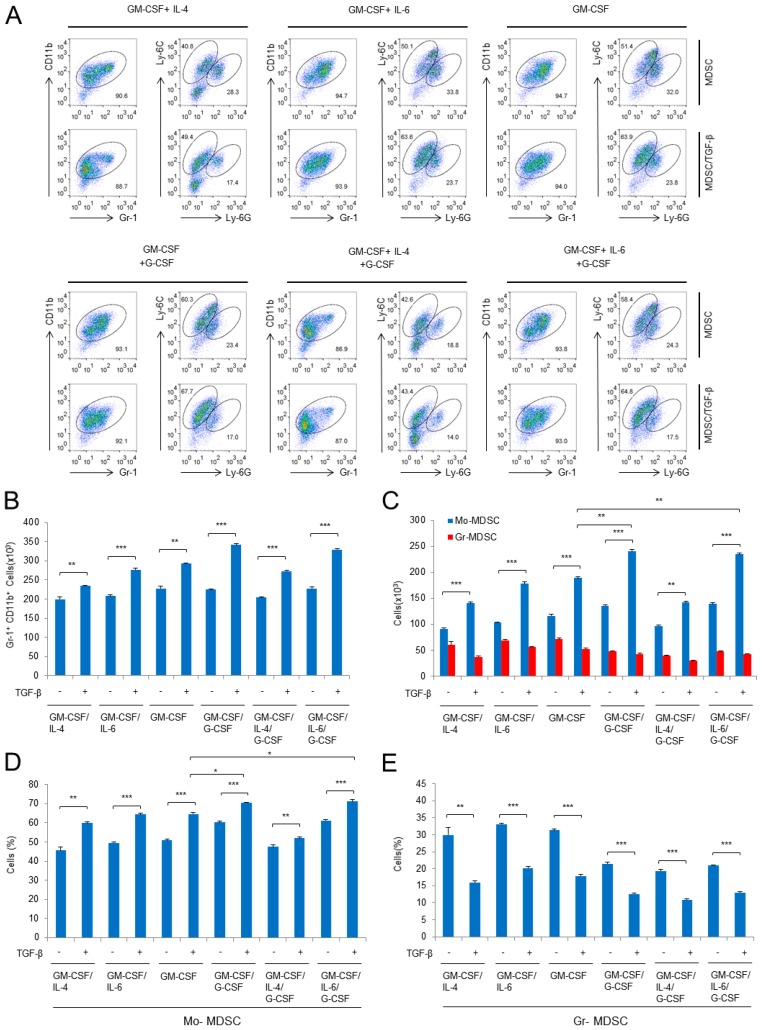
Induction of myeloid-derived suppressor cells (MDSCs) is affected by different combinations of cytokines and by the presence/absence of transforming growth factor-β (TGF-β). (**A**) Flow cytometric analysis of in vitro-induced MDSCs. Bone marrow cells from female mice (6 weeks old) were cultured for 3 days with six different combinations of cytokines; (**B**) cell numbers were analyzed by gating on the Gr-1^+^CD11b^+^ population; (**C**) number of cells in different MDSC subpopulations; (**D**,**E**) percentage of monocytic-MDSCs (Mo-MDSCs) (**D**) and granulocytic-MDSCs (Gr-MDSCs) (**E**). GM-CSF, granulocyte-macrophage colony stimulating factor; IL-4, interleukin-4; IL-6, interlukin-6; G-CSF, granulocyte colony stimulating factor; Data are representative of three independent experiments in triplicate (**B**–**E**), and the results are expressed as the mean ± SD. * *p* < 0.05, ** *p* < 0.01, *** *p* < 0.001 (Student’s *t* test) (**B**–**E**).

In a previous study, we demonstrated that TGF-β can generate subpopulations of MDSCs with increased expression of immunosuppressive factors such as iNOS, TGF-β, IL-10, and Arg1 [23]. In the previous experiments, we used GM-CSF and IL-4 to derive MDSCs from bone marrow cells. It has been reported that G-CSF plays a role in the differentiation of MDSCs [18]. In addition, IL-4 and IL-6 are thought to be positive regulators of immunosuppressive MDSC function [3,18]. Thus, to examine the effect of different cytokines on MDSC induction, we treated mouse bone marrow cells with six different combinations of cytokines: GM-CSF/IL-4, GM-CSF/IL-6, GM-CSF alone, GM-CSF/G-CSF, GM-CSF/G-CSF/IL-4, and GM-CSF/G-CSF/IL-6. GM-CSF was added to all cytokine combinations because it is essential for MDSC differentiation [24]. All cytokine combinations induced Gr-1^+^CD11b^+^ populations. In addition, the Gr-1^+^CD11b^+^ populations could be divided into two subpopulations: Mo-MDSCs and Gr-MDSCs (Figure 1A). TGF-β increased the total MDSC (Gr-1^+^CD11b^+^) numbers in all combinations (Figure 1B) and increased the proportion and number of Mo-MDSCs in the MDSCs (Figure 1C,D); however, the proportion of Gr-MDSCs within the total MDSC population fell, although the numbers did not change by much (Figure 1C,E). The addition of TGF-β to cytokine combinations containing G-CSF greatly affected both the proportion and number of Mo-MDSCs, but it did not appreciably affect cell numbers when IL-4 was added during MDSC differentiation (Figure 1C,D). The data also show that IL-6 did not affect the TGF-β-mediated increase in Mo-MDSCs (Figure 1C,D). Interestingly, in our experiments, G-CSF reduced the proportion of Gr-MDSCs (Figure 1E) even though this cytokine is a survival factor for granulocytes [25]. Overall, these data show that TGF-β is the common factor that drives the increase in Mo-MDSCs (both in proportion and number), but it reduces the proportion of Gr-MDSCs.

### 2.2. Characterization of Immature State of Differentiated MDSCs Induced by Different Cytokine Combinations with or without TGF-β

**Figure 2:**
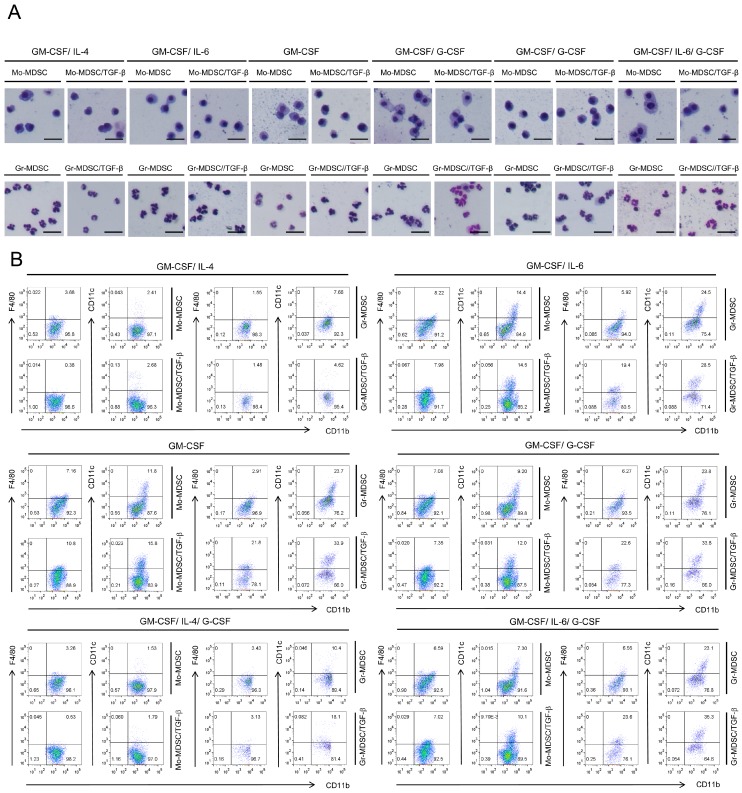
Microscopic phenotype and mature marker expression of the generated MDSCs. (**A**) Wright-Giemsa staining of MDSCs induced by six different cytokine combinations in the presence/absence of TGF-β; The scale bars at the bottom right of images indicate 100 μm. (**B**) Flow cytometry analysis of F4/80 (a marker of mature macrophages) and CD11c (a marker of mature dendritic cells) expression by bone marrow-derived MDSCs differentiated in the presence/absence of TGF-β. GM-CSF, granulocyte-macrophage colony stimulating factor; IL-4, interleukin-4; IL-6, interlukin-6; G-CSF, granulocyte colony stimulating factor; Data are representative of two independent experiments (**A**) or three independent experiments in triplicate (**B**).

First, we examined the morphology of induced MDSCs by Wright-Giemsa staining. Photomicrographs of isolated subpopulations of Gr-1^+^CD11b^+^ cells revealed that Mo-MDSCs had a mononuclear morphology, whereas Gr-MDSCs had a polymorphonuclear morphology, either in the presence or absence of TGF-β (Figure 2A).

The immature status of induced MDSCs was examined by analyzing expression of mature surface markers such as F4/80 and CD11c (Figure 2B and Figure S1), which are markers of macrophages and dendritic cells, respectively; these cells represent mature cell populations induced from myeloid cells, and even fully differentiated functional macrophages or dendritic cells in this culturing system cannot be defined only with those markers. Although there were differences between each cytokine combination, the majority of cells remained immature (F4/80^−^ or CD11c^−^) (Figure 2B). TGF-β treatment of MDSCs induced different responses depending on the cytokine combination used for MDSC differentiation (Figure 2B). During Mo-MDSC differentiation, the data showed that F4/80 expression decreased in the presence of IL-4; under these conditions, TGF-β also decreased F4/80 expression (Figure 2B). However, if IL-4 was absent from the differentiation conditions, F4/80 expression increased and was not reduced by TGF-β (Figure 2B). In addition, IL-4 reduced CD11c expression in the generated Mo-MDSCs, whereas TGF-β had little effect on CD11c expression (Figure 2B). During Gr-MDSC differentiation in the absence of IL-4, TGF-β led to a marked increase in the F4/80^+^ population (Figure 2B). Moreover, omission of IL-4 increased the CD11c^+^ population, which was increased further by the addition of TGF-β (Figure 2B). Overall, the data show that IL-4 is a central factor for maintaining the immature status of MDSCs and that TGF-β drives terminal differentiation of MDSCs in its absence.

### 2.3. MDSCs Derived Using Different Cytokine Combinations Express Immunosuppressive Molecules, the Expression of Which is Enhanced by TGF-β

**Figure 3:**
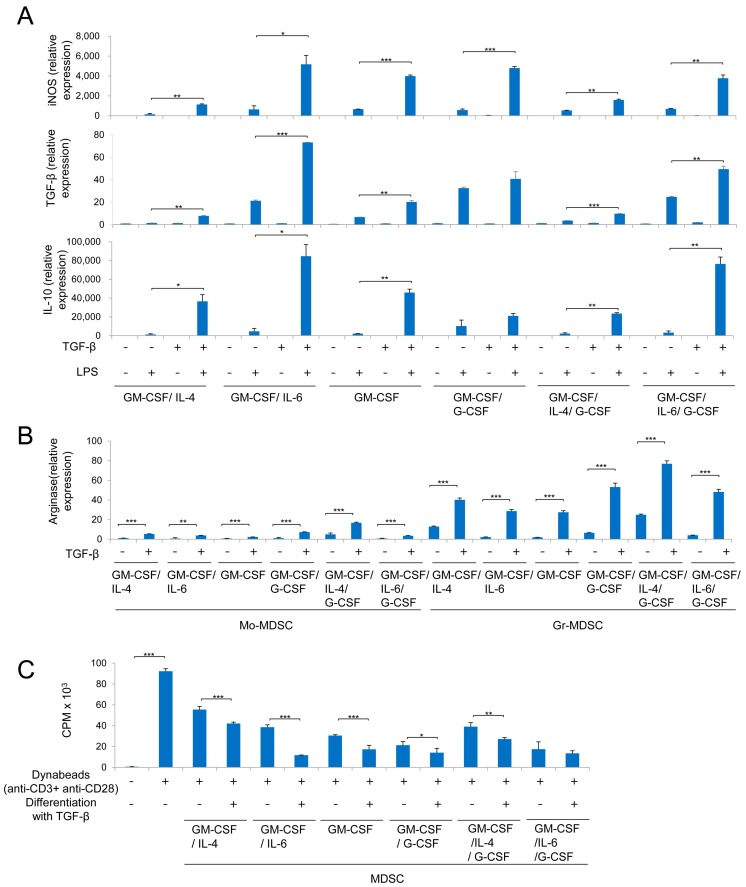
Immunosuppressive functions of the generated MDSCs. (**A**) Quantitative polymerase chain reaction (PCR) analysis of inducible nitric oxide synthase (iNOS), TGF-β, and interleukin-10 (IL-10) expression by isolated Mo-MDSCs in the presence/absence of lipopolysaccharide (LPS) and TGF-β; (**B**) quantitative PCR analysis of Arginase 1 (Arg1) expression by isolated Mo-MDSC and Gr-MDSCs; (**C**) cytokine-induced MDSCs were isolated and cocultured with CD4^+^ T cells. CD4^+^ T cells were then stimulated with anti-CD3- and anti-CD28-coated Dynabeads and labeled with 3H-thymidine. The effector:target ratios (MDSCs: CD4^+^ T cells) were 1:3. GM-CSF, granulocyte-macrophage colony stimulating factor; IL-4, interleukin-4; IL-6, interlukin-6; G-CSF, granulocyte colony stimulating factor; Data are representative of three independent experiments (**A**–**C**), and results are expressed as the mean ± SD. * *p* < 0.05, ** *p* < 0.01, *** *p* < 0.001 (Student’s *t* test).

To investigate the immunosuppressive activity of MDSCs indirectly, we examined expression of iNOS, TGF-β, and IL-10 by Mo-MDSCs, and Arg1 expression by Mo-MDSCs and Gr-MDSCs. Upon lipopolysaccharide (LPS) stimulation, Mo-MDSCs showed increased expression of iNOS, TGF-β, and IL-10 (Figure 3A). IL-4-containing cytokine combinations had a smaller effect on iNOS, TGF-β, and IL-10 expression by Mo-MDSCs than other cytokine combinations in the presence or absence of TGF-β (Figure 3A). However, G-CSF and IL-4 had a marked effect on Arg1 expression by Mo-MDSCs and Gr-MDSCs in the presence and absence of TGF-β (Figure 3B). In addition, the combination of G-CSF plus IL-4 had the greatest effect on Arg1 expression by Mo-MDSCs and Gr-MDSCs. IL-6-containing cytokine combinations had the greatest effect on iNOS, TGF-β, and IL-10 expression by Mo-MDSCs in the presence of TGF-β (Figure 3A). However, G-CSF had the greatest effect on TGF-β and IL-10 expression in the absence of TGF-β (Figure 3A,B). Overall, G-CSF had the greatest effect on expression of immunosuppressive molecules by Mo-MDSCs in the absence of TGF-β, but IL-6 had the greatest effect in the presence of TGF-β. In addition, IL-4 and G-CSF preferentially affected expression of Arg1 by Gr-MDSCs, which was further enhanced by TGF-β (Table 1).

### 2.4. MDSCs Derived Using Different Cytokine Combinations Suppress T Cell Proliferation, an Activity Enhanced by TGF-β

To confirm that indirect immune suppressive properties are linked directly to suppression of T cell proliferation by MDSCs, immune suppression by the differentiated MDSCs was examined. In these experiments, the differentiated MDSCs were cocultured with CD4^+^ T cells, and the T cell proliferation measured. MDSCs suppressed proliferation of CD4^+^ T cells stimulated with anti-CD3 and anti-CD28 antibody-coated beads (Figure 3C). Here, G-CSF had the greatest immunosuppressive effect for MDSCs generated in the absence of TGF-β while IL-6 had the greatest effect in the presence of TGF-β (Table 1). Moreover, for all cytokine combinations, TGF-β led to a significant increase in the immunosuppressive activity of MDSCs for T cell proliferation.

## 3. Discussion

MDSCs suppress T cell activity and proliferation by producing immunosuppressive molecules such as iNOS, TGF-β, IL-10, and Arg1 [3,6]. Mouse models show that MDSCs are defined as Gr-1^+^CD11b^+^ and can be subclassified as Mo-MDSCs or Gr-MDSCs according to the particular Gr-1 epitope: Ly-6G and Ly-6C [3,7]. Under inflammatory conditions, Gr-MDSCs and Mo-MDSCs suppress immune responses via different mechanisms [6]. Gr-MDSCs produce high amounts of Arg1 and ROS, whereas Mo-MDSCs produce NO [3,6]. Previous studies have shown that several cytokines involved in the regulation of MDSCs expansion and function. In those studies, differentiation of MDSCs is affected by G-CSF, IL-4, and IL-6, all of which play a role in the immunosuppressive function of MDSCs [3,18]. In addition, GM-CSF works as a central factor for generation of MDSCs [3,11]. Moreover, those identified cytokines also affect the differentiation and function of MDSCs derived from bone marrow cells [14,26,27]. Thus, testing of cytokines for differentiation and function of bone marrow-derived MDSCs is a valuable strategy to identify the combinational effect of cytokines. In our previous study, we identified TGF-β as a positive regulator for GM-CSF/IL-4-induced MDSCs [23]. However, the effect of TGF-β plus other cytokine combinations on MDSCs differentiation and function has not previously been tested.

Here, we induced differentiation of MDSCs from mouse bone marrow cells using six different cytokine combinations and tested the effect of TGF-β on MDSCs. TGF-β increased the number of Gr-1^+^CD11b^+^ MDSCs and the Mo-MDSC population overall, but decreased the population of Gr-MDSCs under all conditions. This may indicate that TGF-β creates efficient immune regulatory conditions by increasing Mo-MDSCs because Mo-MDSCs possess more potent inhibitory activity than Gr-MDSCs [28]. TGF-β also increased expression of immunosuppressive molecules by Mo-MDSCs and Gr-MDSCs in all conditions. Moreover, suppression activity of MDSCs for proliferation of CD4^+^ T cells was also increased by TGF-β. However, TGF-β had different effects on MDSCs under various differentiation conditions. For example, TGF-β maintained MDSCs in an immature state in the presence of IL-4, but it increased the population of mature MDSCs in the absence of IL-4. In addition, G-CSF was the most important cytokine for the immunosuppressive function of Mo-MDSCs in the absence of TGF-β, but IL-6 greatly enhanced the function of Mo-MDSCs in the presence of TGF-β. Thus, our data reflects the complexity of the relationship between conditions allowing MDSC differentiation and their function under inflammatory conditions.

## 4. Materials and Methods

### 4.1. Mice and MDSC Isolation

Pathogen-free 6-week-old female C57BL/6 mice were purchased from Damul Science (Daejeon, Korea). All mice were kept under specific pathogen-free conditions in the animal care facility at the Gwangju Institute of Science and Technology (GIST). All experiments using mice were approved by the Institutional Animal Care and Use Committee of GIST (GIST-2016-41, approved on 14 October 2016). After differentiation of MDSCs from bone marrow cells, MDSCs were isolated on a Ficoll density gradient (GE Healthcare, Pittsburgh, PA, USA) to remove granulocytes. After removing of the granulocytes, the isolated cells were stained with antibodies against MDSC-specific surface markers, CD11c, and F4/80. The stained cells were isolated using a FACSAria III cytometer (BD Biosciences, Franklin Lakes, NJ, USA). For functional analysis, CD11c and F4/80-negative cells were isolated.

### 4.2. Flow Cytometry

Sorted or in vitro differentiated cells were stained with the indicated antibodies and analyzed using a FACSCanto II (BD Biosciences) or a Guava flow (Millipore, Burlington, MA, USA) cytometer. Live cells were gated by forward and side scatter. Peridinin-chlorophyll proteins/Cy5.5-conjugated anti-mouse CD11b (M1/70; Cat# 45-0112-82), phycoerythrin (PE)-conjugated anti-mouse CD11c (N418; Cat# 12-0114-82), allophycocyanin (APC)-eFluor 780 conjugated anti-mouse F4/80 (BM8; Cat# 47-4801-82), APC-conjugated anti-mouse Ly6C (HK1.4; Cat# 17-5932-82), PE-conjugated anti-mouse Ly6C (HK1.4; Cat# 12-5932-82), and fluorescein isothiocyanate-conjugated Ly6G (RB6-8C5; Cat# 11-5931-82) were purchased from eBioscience (Waltham, MA, USA). Data were analyzed using FlowJo (FlowJo, Ashland, OR, USA) software.

### 4.3. Quantitative Reverse Transcription-Polymerase Chain Reaction

Total RNA was isolated from cell pellets using an RNeasy mini kit (QIAGEN, Hilden, Germany), and first-strand cDNA was synthesized using RT Drymix (Enzinomics, Daejeon, Korea) according to the manufacturer’s instructions. The quantity of mRNA was determined by real-time polymerase chain reaction (PCR; Agilent Technologies, Santa Clara, CA, USA) using TOPreal™ qPCR 2× PreMIX (SYBR Green with low ROX) (Enzynomics). The following primers were used for analyses: mIL-10 (Forward: 5′-GCACTGCTATGCTGCCTGCTCTTACTGA-3′, Reverse: 5′-AGCTTCTCACCCAGGGAATTCAAATGCT-3′), mTGF-β1 (Forward: 5′-CTCCCGTGGCTTCTAGTGC-3′, Reverse: 5′-GCCTTAGTTTGGACAGGATCTG-3′), iNOS (Forward: 5′-CAAATCCTACCAAAGTGACCTGAAA-3′, Reverse: 5′-TACTGTGGACGGGTCGATGTC-3′), Arg1 (Forward: 5′-TCCACCCTGACCTATGTGTCATTT-3′, Reverse: 5′-CGTCTCGCAAGCCAATGTACA-3′). All gene expression levels were normalized using the glyceraldehyde-3-phosphate dehydrogenase gene.

### 4.4. In Vitro Differentiation of Bone Marrow-Derived MDSCs

Bone marrow cells were isolated from C57BL/6 mice, and 5 × 10^5^ cells were cultured in 0.5 mL of RPMI 1640 medium (HyClone, GE Healthcare) supplemented with 10% fetal bovine serum (HyClone, GE Healthcare) containing various combinations of 10 ng/mL GM-CSF (eBioscience, Thermo Fisher Scientific), 10 ng/mL IL-4 (eBioscience, Thermo Fisher Scientific), 10 ng/mL IL-6 (PeproTech, Rocky Hill, NJ, USA), 10 ng/mL G-CSF (PeproTech), and 50 μM 2-Mercaptoethanol (Sigma-Aldrich, St. Louis, MO, USA) with or without 0.2 ng/mL TGF-β as described previously [23]. Cells were cultured in 24-well plates at 37 °C/5% CO_2_. Cells were collected on Day 3 and analyzed by flow cytometry with the appropriate antibodies.

### 4.5. In Vitro Suppression Assays

CD4^+^ T cells (1.5 × 10^5^ cells/well) were isolated from the spleen or lymph nodes by negative selection and cultured in 96-well plates with Dynabeads (Invitrogen, Thermo Fisher Scientific, Waltham, MA, USA) coated with anti-CD3 and anti-CD28 antibodies. MDSCs were isolated using the FACSAria III flow cytometer (BD Biosciences) on the basis of cell surface marker staining. Isolated cells (5 × 10^4^ cells/well for an effector:target ratio of 1:3; 2.5 × 10^4^ cells/well effector:target ratio of 1:6) were cocultured with CD4^+^ T cells. After 54 h of coculture, ^3^H-thymidine (1 μCi/well) was added to each well for an additional 18 h. Cells were harvested on glass filters, and radioactivity was measured using a liquid scintillation counter (Hidex, Turku, Finland).

### 4.6. Statistical Analysis

Statistical analysis was performed using OriginPro 9.1 (OriginLab, Northampton, MA, USA). All data are presented as the mean ± SD. The Student’s *t* test was used to analyze two sets of data.

## 5. Conclusions

Under all cytokine combinations TGF-β increased the proportion of Mo-MDSCs and decreased the proportion of Gr-MDSCs in the total MDSCs. TGF-β also increased expression of immunosuppressive molecules such as iNOS, TGF-β, IL-10, and Arg1, and increased MDSC-mediated suppression of CD4^+^ T cell proliferation. Thus, even though this cytokine had different effects on maturation and immunosuppression under a variety of differentiation conditions, our data suggest that TGF-β plays a positive role in MDSC expansion and function. Furthermore, our data may be useful for an inflammatory disease-specific cell therapeutic approach using in vitro differentiated MDSCs. Thus, this report is helpful for development of optimized MDSCs differentiation protocol for an inflammatory disease-specific cell therapy.

## Figures and Tables

**Table 1:** Summary of MDSCs characteristics induced with six cytokine combinations with or without TGF-β.

Characteristics		Mo-MDSCs												Gr-MDSDCs											
		GM-CSF/IL-4		GM-CSF/IL-6		GM-CSF		GM-CSF/G-CSF		GM-CSF/IL-4/G-CSF		GM-CSF/IL-6/G-CSF		GM-CSF/IL-4		GM-CSF/IL-6		GM-CSF		GM-CSF/G-CSF		GM-CSF/IL-4/G-CSF		GM-CSF/IL-6//G-CSF	
TGF-β		−	+	−	+	−	+	−	+	−	+	−	+	−	+	−	+	−	+	−	+	−	+	−	+
Population change	Percentage	1	5	2	7	2	7	6	9	1	3	6	10	8	3	10	5	9	3	5	1	4	1	4	1
	Number	1	3	1	5	2	6	3	10	1	4	3	10	7	2	9	6	9	5	5	3	3	1	10	3
Immature Population	F4/80^−^	7	10	3	3	4	1	4	4	7	9	4	4	9	10	8	2	9	1	8	1	9	9	7	1
	CD11c^−^	9	8	1	1	3	1	4	3	10	9	5	4	9	10	4	2	4	1	4	1	8	5	4	1
Suppression Marker	Arginase	1	3	1	3	1	2	1	4	3	10	1	3	2	5	1	4	1	3	1	7	3	10	1	6
	iNOS	1	3	2	10	2	7	1	9	1	5	2	7												
	TGF-β	1	1	3	10	1	3	4	5	1	2	3	7												
	IL-10	1	4	1	10	1	5	2	4	1	5	1	9												

MDSCs, myeloid-derived suppressor cells; Mo-MDSCs, monocytic MDSCs; Gr-MDSCs, granulocytic MDSCDs, GM-CSF, granulocyte-macrophage colony stimulating factor; IL-4, interleukin-4; IL-6, interlukin-6; G-CSF, granulocyte colony stimulating factor; TGF-β, transforming growth factor-β; iNOS, inducible nitric oxide synthase; IL-10, interleukin-10. Numbers indicate levels from the data. Level 10 is reserved for the highest value and level 1 is reserved for the lowest value in the data. The relative level was indicated in the table.

## Supplementary Materials

Supplementary materials can be found at http://www.mdpi.com/1422-0067/19/3/869/s1.

## Abbreviations

MDSCs	myeloid-derived suppressor cells
Gr-MDSC	granulocytic-MDSC
Mo-MDSC	monocytic-MDSC
ROS	reactive oxygen species
Arg1	arginase 1
NO	nitric oxide
iNOS	inducible nitric oxide synthase
TGF-β	transforming growth factor-β
IL-10	interleukin-10
CSF	colony-stimulating factors
GM-CSF	granulocyte-macrophage CSF
M-CSF	macrophage CSF
G-CSF	granulocyte CSF
STAT3	signal transducer and activator of transcription 3
IL-4Rα	interleukin-4 receptor α
IL-4	interleukin-4
IL-6	interleukin-6
LPS	lipopolysaccharide
PE	phycoerythrin
APC	allophycocyanin
PCR	polymerase chain reaction
