# Supplementary material for: Characterization of Multiple Cytokine Combinations and TGF-β on Differentiation and Functions of Myeloid-Derived Suppressor Cells
Source: Int J Mol Sci. 2018 Mar 15;19(3):869. doi: 10.3390/ijms19030869 (PMC5877730; doi:10.3390/ijms19030869)
Supplement: Supplementary file 1 [file ijms-19-00869-s001.pdf]

Characterization of multiple cytokine combinations and TGF-β on differentiation and functions of myeloid-derived suppressor cells

Cho-Rong Lee†, Wongeun Lee†, Steve K. Cho and Sung-Gyoo Park*

School of Life Sciences, Gwangju Institute of Science and Technology (GIST), Gwangju 61005, Republic of Korea

*Correspondence: sgpark@gist.ac.kr; Tel.: +82-62-715-2511

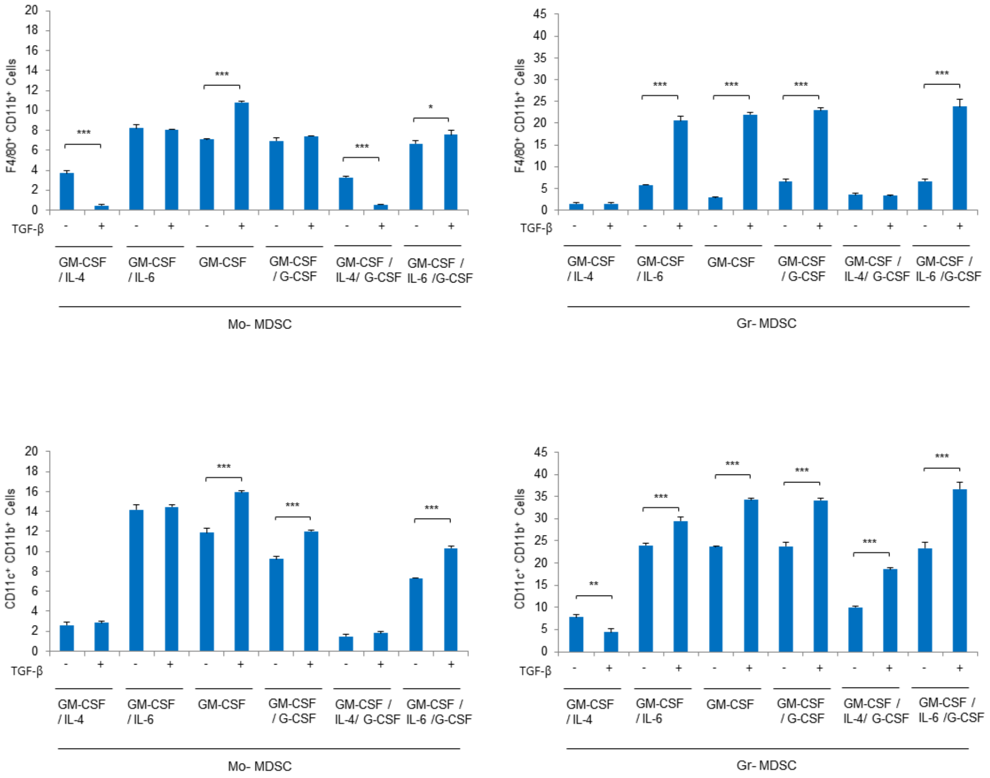

Supplementary Figure 1. Mature marker expression of the generated MDSCs. Summary of flow cytometry analysis of F4/80 (a marker of mature macrophages) and CD11c (a marker of mature dendritic cells) expression by bone marrow-derived MDSCs differentiated in the presence/absence of TGF-β. Data are representative of three independent experiments in triplicate, and the results are expressed as the mean ± SD. *$p < 0.05$, **$p < 0.01$, *** $p < 0.001$ (Student's t test).
